# Proximity ligation scaffolding and comparison of two *Trichoderma reesei* strains genomes

**DOI:** 10.1186/s13068-017-0837-6

**Published:** 2017-06-12

**Authors:** Etienne Jourdier, Lyam Baudry, Dante Poggi-Parodi, Yoan Vicq, Romain Koszul, Antoine Margeot, Martial Marbouty, Frédérique Bidard

**Affiliations:** 1 IFP Energies nouvelles, 1 et 4 Avenue de Bois-Préau, 92852 Rueil-Malmaison, France; 2 Groupe Régulation Spatiale des Génomes, Department Genomes and Genetics, Institut Pasteur, 75015 Paris, France; 3 UMR 3525, CNRS, 75015 Paris, France

**Keywords:** *Trichoderma reesei*, Genome assembly, Hi-C, GRAAL, Centromere, Karyotype, Translocation, Chromosomal contact, Chromosome conformation capture

## Abstract

**Background:**

The presence of low complexity and repeated regions in genomes often results in difficulties to assemble sequencing data into full chromosomes. However, the availability of full genome scaffolds is essential to several investigations, regarding for instance the evolution of entire clades, the analysis of chromosome rearrangements, and is pivotal to sexual crossing studies. In non-conventional but industrially relevant model organisms, such as the ascomycete *Trichoderma reesei*, a complete genome assembly is seldom available.

**Results:**

The chromosome scaffolds of *T. reesei* QM6a and Rut-C30 strains have been generated using a contact genomic/proximity ligation genomic approach. The original reference assembly, encompassing dozens of scaffolds, was reorganized into two sets of seven chromosomes. Chromosomal contact data also allowed to characterize 10–40 kb, gene-free, AT-rich (76%) regions corresponding to the *T. reesei* centromeres. Large chromosomal rearrangements (LCR) in Rut-C30 were then characterized, in agreement with former studies, and the position of LCR breakpoints used to assess the likely chromosome structure of other *T. reesei* strains [QM9414, CBS999.97 (1-1, *re*), and QM9978]. In agreement with published results, we predict that the numerous chromosome rearrangements found in highly mutated industrial strains may limit the efficiency of sexual reproduction for their improvement.

**Conclusions:**

The GRAAL program allowed us to generate the karyotype of the Rut-C30 strain, and from there to predict chromosome structure for most *T. reesei* strains for which sequence is available. This method that exploits proximity ligation sequencing approach is a fast, cheap, and straightforward way to characterize both chromosome structure and centromere sequences and is likely to represent a popular convenient alternative to expensive and work-intensive resequencing projects.

**Electronic supplementary material:**

The online version of this article (doi:10.1186/s13068-017-0837-6) contains supplementary material, which is available to authorized users.

## Background

*Trichoderma reesei* is one of the main industrial enzyme producers [1]. This Ascomycota naturally produces a full set of lignocellulosic biomass degrading enzymes, and carries high stakes for the food, textile, and bioenergy industries. Over the years, the enzyme production has been boosted through cycles of random mutageneses, with highly performing strains secreting up to 100 g L^−1^ of the natural enzyme mix [2]. *T. reesei* is also increasingly used as a versatile heterologous protein producer [3, 4]. In contrast to its industrial interest, the genetic tools available in *T. reesei* have developed at a slower pace than in other model filamentous fungi such as *Neurospora crassa* partly because of the small research community sometimes constrained by industrial confidentiality imperatives. In addition, until recently [5], neither sexual crossings nor any annotated karyotype were available for this fungus.

*Trichoderma reesei*, described from a single wild-type isolate called QM6a, was believed to be devoid of a sexual cycle, whereas its teleomorph, *Hypocrea jecorina*, undergoes an heterothallic sexual cycle involving *MAT1*-*1* and *MAT1*-*2* loci [6]. The identification of a *MAT1*-*2* locus in the QM6a followed by a sexual crossing with a natural isolate of a *MAT1*-*1* type resulted in fertilized stromata and mature ascospores [5]. QM6a and its derivatives (of which QM9414, NG14, Rut-C30 [7]) are female sterile but male fertile and could nevertheless be crossed with a *MAT1*-*1* natural isolate acting as female partner, paving the way to the development of sexual crossing tools to generate genetic diversity, genetic cleanup, and strain improvement. Several groups have since built on this original finding by characterizing the receptor/pheromone system [8], uncovering the causes for female sterility [9] and studying meiosis [10] in this species. The latter study have demonstrated the biotechnological interest of crossings different industrial strains but also underlined their limits by pointing at the presence of segmental aneuploidies and chromosome rearrangements resulting in non-viable ascospores.

Chromosomal rearrangements in mutagenized *T. reesei* strains have been first described in the nineties [11, 12]. The karyotypes of industrial strains descending from the parental QM6a strain by several rounds of random mutagenesis displayed massive rearrangements, as revealed by pulse-field gel electrophoresis (PFGE). However, the relatively low resolution of the PFGE technique for chromosomes of similar sizes led to discrepancies between the original studies, and the precise karyotypes of the strains remained elusive. Years later, the draft sequence of the QM6a strain genome was released as a set of 89 scaffolds [13]. Subsequent efforts to obtain genomic wide information of other strains of the same lineage used either genome walking [14], oligonucleotide arrays [15], or short-reads sequencing platform [16–19] but did not improve the assembly. Even though the positions of chromosomal breakpoints were identified for several derivative strains [15], the impact on the chromosomal structure was difficult to assess because of the lack of a complete assembly. In addition, centromeres and telomeres positions remained unknown, as these regions are typically difficult to sequence and assemble because of their low complexity and, for centromeres, the lack of universal conserved sequence patterns. However, reaching at a full genome scaffolds remains an important goal for these model fungi [20]. In the case of *T. reesei*, getting the sequence and exact position of centromeres would provide invaluable information for the emerging sexual crossing field in this species. More broadly, information on centromeres in filamentous fungi remains sparse, and these sequences would bring interesting highlights onto their evolution and metabolism [21].

Using chromosome conformation capture data (3C; or also dubbed proximity ligation data) [22] and the homemade program GRAAL (Genome Re-Assembly Assessing Likelihood from 3D), our groups recently published the first proximity ligation scaffolding of an incomplete eukaryotic genome sequence. The 89 scaffolds of the *T. reesei* QM6a strain were re-scaffolded into seven chromosomes [23, 24]. In addition, the “Rabl” structure of chromosomes in fungi nuclei, where centromeres are clustered together at the microtubule organization center (spindle pole body in yeast), generates contacts enrichment between these sequences. When quantified, we also showed that the signal resulting from these 3D contacts allows the identification of centromere positions [25]. Although the QM6a contact map displayed such signal, we did not at the time characterize precisely these sequences. The published sequence from this past work was not thoroughly integrated within the JGI reference genome database, though it was nevertheless exploited in independent analyses by others [26].

Here, we provide an updated version of the QM6a chromosome scaffolding using an extra polishing step after GRAAL output. GRAAL is a scaffolding pipeline that processes pre-assembled contigs; as a result, the resulting assembly displays the same sequence as in the original genome. We also exploited the 3C contact map to identify the position and sequences of the QM6a centromeres [25], providing insight about *T. reesei* centromeres. The same pipeline was applied to the QM6a-derived strain Rut-C30, resulting in a genome scaffold in perfect agreement with previously identified chromosomal rearrangements between the two genomes [14, 15]. This result prompts us to put forward predictive karyotypes for several other *T. reesei* strains and to discuss the impact of such karyotypes on the emergence of segmental aneuploidy during crossing experiments [10].

## Results

### Improved QM6a chromosome assembly

The *T. reesei* QM6a genome was scaffolded into superscaffolds using the reference assembly from Martinez et al. [13] and the chromosome contact reads from Marie-Nelly et al. [23]. Scaffolding was performed using the latest version of GRAAL [27] run for 100 iterations. The scaffolding remains nearly identical to the one published previously, with seven superscaffolds matching the seven chromosomes [23]. Again, a fraction (0.5%) of the original assembly was not included in the superscaffolds, as a result of low 3C sequencing coverage (lack of restriction sites and/or highly divergent GC content could account for such low coverage).

**Fig. 1:**
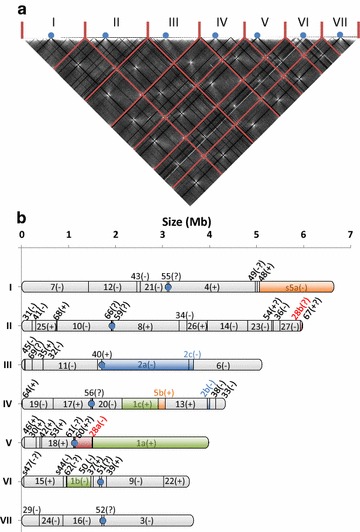
*T. reesei* scaffold reassembly in seven chromosomes. *T. reesei* scaffolds from the JGI reference genome have been reassembled using chromosomal conformation capture (3C) sequencing data. **a** Contact matrix resulting from GRAAL reassembly. *Red bars* indicate the boundaries of the seven chromosome; centromere positions are represented by *blue dots*. **b** Order and orientation of the reassembled scaffolds in the seven chromosomes. Orientation uncertainties are noted with a question mark. Scaffolds 1, 2, 5, and 28 that were misassembled in the reference genome are shown in *green*, *blue*, *orange*, and *red*, respectively. Centromere positions are represented by *blue dots*

Because the resolution of the GRAAL scaffolding is limited by the distribution of restriction sites along the chromosome and the read coverage, a manual curation was necessary to complete the assembly. This step includes reinserting missing scaffold fragments, checking telomere repeats’ orientations, and slightly shifting split locations to remain consistent with the presence of N gaps in the reference genome (see “Methods”). The resulting QM6a GRAAL scaffolding is fully consistent with the JGI reference genome, containing exactly the same sequences than original scaffolds. 65 scaffolds, comprising 99.5% of the genome, were scaffolded along seven chromosomes (Fig. 1). 22 scaffolds, representing 0.5% of the genome, were either too small (not enough restriction sites along their sequences) or insufficiently covered (not enough reads during 3C library sequencing) to be scaffolded within the chromosomes. We did not sequence the gaps between reassembled scaffolds, and instead 100 Ns were intercalated between scaffolds as a marker of GRAAL scaffolding position. Additional sequencing work would therefore be required to reach a final fully continuous genomic sequence. In a simultaneous and independent study from Ting-Fang Wang’s team, a QM6a resequencing was performed (Wan-Chen Li et al. personnel communication). We agreed on the chromosome nomenclature (order by decreasing size, numbering with Roman numerals, and orientation with left arm shorter than right arm) so as our works are consistent.

Most scaffolds from the reference genome remained intact in the reassembly (in gray Fig. 1b). However, four scaffolds (1, 2, 5, and 28) were misassembled in the reference genome and were split by GRAAL into several segments in the new scaffolding (Fig. 1b) [23]. The split location of scaffold 28 and its reassembly with scaffolds 27 and 36 is consistent with deep sequencing of the CBS999.97 (1-2, *wt*) strain, whose genome is similar to QM6a [10]. We previously suggested that a fragment of scaffold_9 (≈1020–1045 kb) containing the ribosomal DNA units was duplicated on chromosome VI [23]. However, we were not able to determine the precise number of copies (probably three or four) and the exact sequence to assemble these copies, and we preferred to leave the exact sequence of scaffold_9 as in the JGI reference genome. Therefore, chromosome VI is in fact longer than chromosome VII (Wan-Chen Li et al. personnel communication).

Table 1 shows statistics on chromosome sizes, number of genes, and gene densities. Gene density in *T. reesei* is much more uniform than suggested [26], ranging from 0.26 to 0.28 genes per kb. Additional files 1, 2, 3, contain details on this reassembly (scaffold assembly, final sequence, gene annotation).

**Table 1:** Size (bp) , number of genes, and gene density (nb of genes per kb) of *T. reesei* QM6a chromosomes

Genetic element	Size	Number of genes	Gene density
Chromosome I	6,647,935	1817	0.27
Chromosome II	5,980,447	1701	0.28
Chromosome III	5,112,650	1336	0.26
Chromosome IV	4,337,413	1162	0.27
Chromosome V	3,979,336	1092	0.27
Chromosome VI	3,567,305	983	0.28
Chromosome VII	3,660,386	1022	0.28
Unassembled scaffolds	163,868	16	
Total	33,449,340	9129	

Gene annotation was based on the JGI Filtered Models set of genes

### Centromere locations

Fungi chromosome organization typically follows a “Rabl” pattern, with the centromeres colocalizing at the microtubule organizing center. For instance, the strong *trans* contact signal between centromeres of *Saccharomyces cerevisiae* reflects this organization, resulting in discrete dots over the contact map of this species [28]. We have previously shown that centromere–centromere 3D contacts can be used to infer the positions of these regions along the 1D sequence [25]. The bright dots clearly visible in the contact map of the *T. reesei* QM6a genome unveiled a clear Rabl organization (Fig. 1a), pointing at the centromeric regions in this species, and allowing us to identify their positions along the seven chromosomes (Table 2). These centromere signatures pointed at a set of 11 small scaffolds ranging in size from 11 to 43 kb (total length of 270 kb). Three of them (57, 58, and 65) could not be assigned to specific chromosomes (they are part of the 22 unassembled scaffolds), but the eight others were scaffolded within six of the seven chromosomes. For chromosome III, the centromere signature was found at the frontier between scaffolds 2 and 40, but we were not able to identify which centromere scaffold among scaffolds 57, 58, or 65, should be reassembled at this place. The centromeres of chromosome I, VI, and VII are metacentric, whereas the four others (chromosomes II to V) are submetacentric, with the longer (right) arm of the chromosome roughly twice as long as the shorter (left) arm.

**Table 2:** *T. reesei* QM6a centromeres

Chr	Location on chr (Mb)	Between scaffolds	Scaffolds involved	Size (kb)	%AT	Nb of genes (gene IDs)
Scaffolds with centromere signature reassembled in chromosomes						
chr I	3.12	21(−) and 4(+)	55	34	77.9	4 (112,674, 112,675, 112,676, 112,677)
chr II	1.93	10(−) and 8(+)	66 + 59	30	70.0	3 (71,146, 43,199, 42,942)
chr III	1.71	40(+) and 2a(−)	Unknown			
chr IV	1.48	17(+) and 20(−)	56	32	74.2	2 (112,678, 112,679)
chr V	1.12	18(+) and 28a(−)	60 + 61	32	77.0	1 (112,683)
chr VI	1.67	37(+) and 39(+)	51	43	76.3	2 (112,649, 73,103)
chr VII	1.73	16(−) and 3(−)	52	41	76.7	1 (112,651)
Other scaffolds with centromere signature but not reassembled						
			57	26	76.6	0
			58	21	78.7	3 (112,680, 112,681, 112,682)
			65	13	81.7	1 (112,689)

Chromosomal contact data were used to identify the location of the centromeres on the chromosomes. Centromeres were all identified in small scaffolds, not in the middle of well-assembled scaffolds

### AT content in centromeres

The average AT content of these centromere scaffolds is 76%, a much higher value than the average AT genomic content (48% [13]), and consistent with other fungal centromeres [21]. We checked whether this high AT content was specific to centromeres or telomeres by looking for AT-rich regions (%AT >65% and length >4 kb) in the whole genome. In addition to the 270 kb centromere scaffolds, 776 kb AT-rich regions were identified over the genome (98 kb at telomeres; 604 kb split over 72 intra-chromosomal regions; 74 kb in 12 unassembled scaffolds). Most AT-rich regions were positioned at the end of scaffolds, which may explain the previous assembly failures.

### Genes in and around centromeres

Seventeen genes were annotated in these 11 scaffolds but all seems to be dubious Coding DNA Sequences (CDS) with many or very large introns, and their products are all annotated as putative proteins of unknown function. Using previously generated RNA-Seq data ([29] and Pirayre et al. to be published), we checked for transcription in these centromere scaffolds and we did not observe any transcription event. So it seems that most probably no gene is present on these scaffolds involved in *T. reesei* centromeres. Function enrichment analysis in close proximity to the centromeres (in a 50-kb window around centromeres) revealed significant enrichments in genes involved in nucleosome assembly (5 genes annotated with the GO term GO:0006334) and in genes linked to the respiratory chain (15 genes in the metabolic pathways of coenzyme Q biosynthesis, adenosine ribonucleotides de novo biosynthesis, and respiration). We can only make assumptions on the significance of this finding, but it could be that their presence in a zone of pericentric repression of crossover is a sign of their importance for the organism robustness and fitness [30]. Interestingly, the CenH3 (centromere-specific histone H3) encoding gene 57870 (orthologue of *N. crassa* NCU00145 and *S. cerevisiae* CSE4) was found on chromosome I at only 30 kb from the centromere (0.5% of the chromosome length). This feature is not conserved in other species, for example, *Schizosaccharomyces pombe* Cnp1 is found at 1.93 Mb from the centromere [31].

### Inverted repeats

Although aware that centromeres were not fully assembled, we checked their sequences for homologies or repeats. We did not observe any sequence homology between centromere regions, which is consistent with the now accepted finding that most centromeres are epigenetically and not genetically maintained [32]. Remarkably, in four cases [scaffolds 51 (chr. VI), 56 (chr. IV), 57 and 58], we observed an inverted repeat structure with a central core region of 1–2 kb surrounded by an inverted repeat of 2.5–5 kb, which is quite similar to the centromere structure of *S. pombe* [31, 33, 34], *Candida albicans* [35], *Candida tropicalis* [36], and *Komagataella phaffii* (formerly *Pichia pastoris*) [37]. Details on this observation are available on Additional file 4 but a complete study on *T. reesei* centromeres structure would require a full assembly, and chromatin immunoprecipitation sequencing experiments.

### Rut-C30 chromosome assembly

In order to get a chromosomal map of *T. reesei* Rut-C30, a 3C library of the Rut-C30 strain was generated, sequenced, and the resulting reads exploited to rescaffold the QM6a genome. Although a genomic sequence was available for *T. reesei* Rut-C30 strain [17], the JGI reference sequence of *T. reesei* QM6a strain was used to demonstrate that the approach could be applicable to any other non-sequenced strain, even if significant chromosomal rearrangements are expected.

**Fig. 2:**
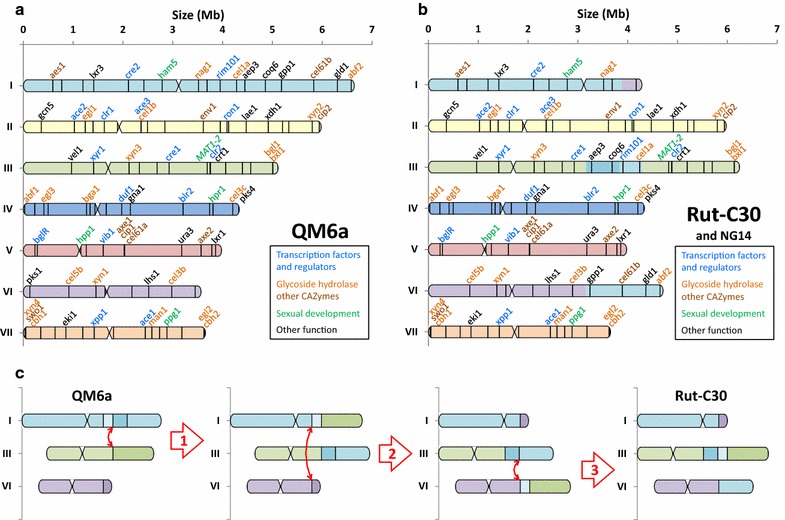
Chromosome maps of *T. reesei* QM6a and Rut-C30 strains. Chromosome maps of *T. reesei* QM6a (**a**) and Rut-C30 and NG14 (**b**) strains were identified by reassembly of the JGI reference genome using 3C sequencing data for each strain. For Rut-C30 map, the *colors* of chromosome fragments are consistent with their *colors* in QM6a map to clearly show chromosomal rearrangements. Some emblematic genes were chosen along the sequence to be used as location markers (list available in Additional file 6). The Rut-C30 85 kb deletion event on chr. VI is shown by the lack of pks1 gene. Centromere locations are shown by restricted width. **c** Possible scenario (among others) from QM6a to Rut-C30. Translocations are numbered according to Table 3

GRAAL identified three chromosomal translocations and one large deletion (Table 3) present in Rut-C30 compared to the QM6a, in agreement with previous work [14, 15]. By design, and as stated before, GRAAL identifies rearrangement events with a precision limited by the sequencing coverage and the restriction pattern of the region (in this case, a couple of dozens of kb; “Methods”). Besides the rearrangements listed in Table 3, the two genome assemblies of Rut-C30 and QM6a were compared and did not present major differences: the reordering of the scaffolds not involved in chromosomal rearrangements (including the splitting of the misassembled scaffolds 1, 2, 5, and 28), as well as centromere positions, were fully consistent between the two assemblies (the Rut-C30 reassembly is available in Additional file 5). The fully scaffolded genomes of these two strains can then be compared in an attempt to have a better understanding of the evolutionary trajectories of the evolved Rut-C30 genome (Fig. 2). Different scenarios are possible from QM6a to Rut-C30, depending on the order of occurrence of the three translocation events, leading to the same chromosome structure. One possible scenario is shown Fig. 2c.

**Table 3:** Translocation and large deletion events found in GRAAL reassembly of *T. reesei* Rut-C30 with respect to QM6a

Translocation	Location on scaffolds (this study)	Location on scaffolds [15]	Mapping on QM6a chromosomes
n° 1	scaffold_2: 556 ± 22 kb	scaffold_2: 546,703 bp	chr III: 3,166,447
	scaffold_4: 1,197 ± 25 kb	scaffold_4: 1,204,862 bp	chr I: 4,342,096
n° 2	scaffold_4: 750 ± 27 kb	scaffold_4: 748,277 bp	chr I: 3,885,511
	scaffold_22: 138 ± 31 kb	scaffold_22: 139,515 bp	chr VI: 3,165,364
n° 3	scaffold_22: 138 ± 31 kb	scaffold_22: 139,476 bp	chr VI: 3,165,325
	scaffold_48: 0 ± 35 kb	scaffold_48: 1667 bp	chr I: 5,018,020

Large deletion	Location on scaffold (this study)	Location on scaffold [14]	Mapping on QM6a chromosome
85-kb deletion	scaffold_15: 0–85 ± 25 kb	scaffold_15: 1,555–86,603	chr VI: 52,198–137,246

Newly acquired 3C-seq data of *T. reesei* Rut-C30 strain were used to reassemble the reference genome. Comparison with QM6a reassembly allowed the identification of three chromosomal translocations and one large deletion. The position of these rearrangements is consistent with former work [14, 15]

The three translocations resulted finally in the right arm of chromosome I (3′ end of scaffold 48 and main fragment of scaffold 5: 1.63 Mb and 442 genes in total), to be swapped with the right arm of chromosome I (3′ end of scaffold 22: 402 kb and 114 genes). But also in two fragments of chromosome I (one with a fragment of scaffold 4, and the other one with another fragment of scaffold 4, scaffold 49, and a small fragment of scaffold 48) to be inserted head to foot in the middle of the chromosome V (1.13 Mb and 310 genes in total for both fragments). Therefore, the whole sequence of chromosome III is still found on chromosome III. The 85 kb deletion is closed to the telomeric region of chromosome VI and therefore one of its flanking is an AT-rich region as previously described [14]. Except for the breakpoint chr I: 5,018,020 localized inside an AT-rich region, the %GC in a 1-kb window around the breakpoints displayed a similar or higher level than in the genome. The four events listed in Table 3 for Rut-C30 strain were already present in its ancestor NG14 [14, 15], so the chromosome structure of NG14 strain is most likely identical to Rut-C30 chromosome structure (Fig. 2b). The chromosomal rearrangements identified previously by the CGH array stud [15] and a genomics analysis [17] are in line with the contact map results obtained in this study. So it should be possible to reconstitute the karyotypes of other *T. reesei* strains for which this kind of information is available.

### Inferring the chromosome structure of other *T. reesei* strains

**Fig. 3:**
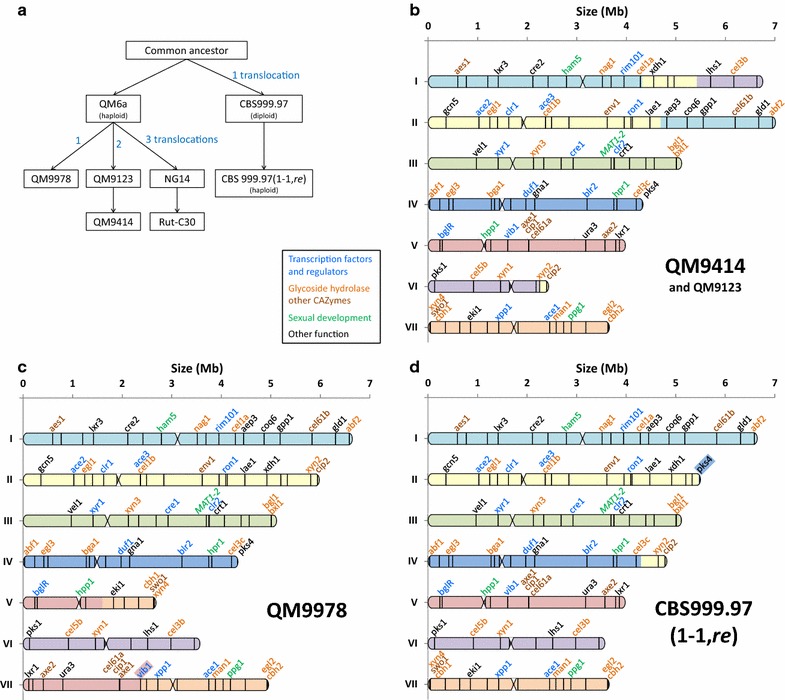
Genealogy and likely chromosome structure of various *T. reesei* strains. Translocation breakpoints (Table 4) and QM6a chromosome assembly (Fig. 2) were used to infer the likely chromosome structure of various *T. reesei* strains. Chromosome fragment *colors* and marker genes are consistent to QM6a map (Fig. 2: chromosome maps of *T. reesei* QM6a and Rut-C30 strains). **a** Genealogy of *T. reesei* strains. **b** Likely chromosome map of QM9414 and QM9123. **c** Likely chromosome map of QM9978. **d** Likely chromosome map of CBS999.97 (1-1, *re*)

We then confronted the QM6a chromosome structure with translocation events characterized in other *T. reesei* strains to reconstitute their expected karyotypes. Table 4 shows translocation breakpoints for the QM9414, QM9123 [15], CBS 999.97(1-1, *re*) [10], and QM9978 (Ivanova et al. to be published) strains, and their mapping on QM6a chromosomes. For each strain, the possible chromosome structure was assessed from these translocation events (Fig. 3). In QM9414 strain (Fig. 3b), two translocations involved chromosomes I, II, and VI, with among others, one fragment of chromosome II and one fragment of the VI being translocated onto chromosome I. In QM9978 (Fig. 3c), a reciprocal translocation event involved chromosomes V and VII, with the chromosome V breakpoint positioned 1.6 kb upstream the gene 54675 that encodes for the transcription factor VIB1. This rearrangement, by modifying the transcription of this gene, is responsible of the cellulase-negative phenotype of this strain (Ivanova et al. to be published). Finally, the translocation event in the diploid strain CBS 999.97 involved chromosomes II and IV, and resulted in the isolation of haploid strains either of WT or recombinant (*re*) karyotypes (Fig. 3d) [10].

**Table 4:** Translocation breakpoints of various *T. reesei* strains genomes

	Translocation breakpoints	Location on QM6a scaffolds [15]	Mapping on QM6a chromosomes
QM9414 & QM9123	n°1	scaffold_4: 1,190,139	chr I: 4,327,373
		scaffold_14: 118,472	chr II: 4,693,330
	n°2	scaffold_9: 787,779	chr VI: 2,237,971
		scaffold_27: 140,159	chr II: 5,788,998
CBS 999.97 (1-1, *re*)	Resulting in D-segment	scaffold_36: 54,323	chr II: 5,441,472
	Resulting in L-segment	scaffold_33: 33,249	chr IV: 4,304,165
QM9978	n°1	scaffold_1: 96,633	chr V: 1,604,851
		scaffold_16: 631,551	chr VII: 1,076,804

Translocation breakpoints were mapped on the superscaffolds generated by GRAAL

### Essentiality of the chromosomes fragments

When crossing CBS999.97 (1-1, *re*) with either CBS999.97 (1-2, *wt*) or QM6a, Chuang et al. showed that L-segment aneuploidy (containing 11 genes in our reassembly) is not lethal but results in a “white spore” phenotype because of the loss of the polyketide synthase 4 gene (tpks4, gene ID 82208) located on this segment [10]. On the other hand, loss of the D-segment (containing 167 genes in our reassembly) is not viable, most probably because essential genes are present on this segment. For each of the translocations listed in Tables 3 and 4, we computed the length and number of genes of the resulting chromosome fragments, from the breakpoint to the telomere (or to the next breakpoint in the case of QM9414 chromosome II and Rut-C30 chromosome I) (Table 5). Then we looked for essential genes in each of these chromosome fragments to verify whether their loss will be lethal or not.

**Table 5:** Statistics on chromosome fragments

Strain	Chromosome	Fragment size (kb)	Nb of genes
CBS 999.97 (1-1, *re*)	chr II => chr IV (D-segment)	539	167 genes
	chr IV => chr II (L-segment)	33	11 genes
QM9414 & QM9123	chr I => chr II	2321	634 genes
	chr II => chr I	1096	322 genes
	chr II => chr VI	192	63 genes
	chr VI => chr I	1329	369 genes
QM9978	chr V => chr VII	2374	644 genes
	chr VII => chr V	1077	290 genes
Rut-C30	chr I => chr III	1133	309 genes
	chr I => chr VI	1630	442 genes
	chr III => chr III	1976	485 genes
	chr VI => chr I	402	114 genes

For each of the breakpoint described in Tables 3 and 4, the size and number of genes of the resulting chromosome fragment (from the breakpoint to the telomere or to the next breakpoint) were calculated. The only dispensable fragment is the L-segment described in CBS999.97 (1-1, *re*) [10]

In QM9414 strain, the fragment of chromosome II which has been translocated to chromosome VI contains only 63 genes, in which the ribosomal protein RPS24 (gene ID 81713) has been shown to be essential for 40S ribosomal subunit assembly in HeLa cells [38]. In Rut-C30 strain, the fragment of chromosome VI which has been translocated on chromosome I contains 114 genes, among which the acetyl-CoA carboxylase (geneID 81110) is presumably essential (its orthologue cut6 is essential in *S. pombe* [39]). All other chromosome fragments listed on Table 5 contain at least 290 genes. Assuming 18.7% of essential genes as in *S. cerevisiae* [40], the probability that these fragments do not contain an essential gene is below 10^−26^. Therefore, the only translocated fragment which is not essential is the small previously described CBS999.97 (1-1, *re*) L-segment [10].

### Inferring lethal segmental aneuploidy in F1 progenies

**Fig. 4:**
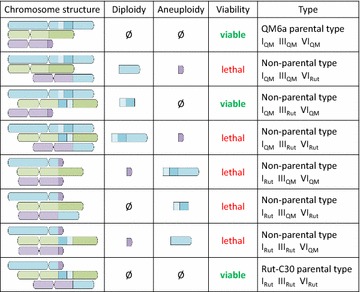
Possible chromosome structures in F1 progeny resulting from a crossing between a *MAT1*-*1* female fertile QM6a strain and Rut-C30 strain. Using the chromosome structure of QM6a and Rut-C30 strains, we enumerated the possible chromosome structures in F1 progeny (only chromosomes I, III, and VI are shown here with *colors* consistent to Fig. 3c). For each possible structure, the fragmental diploidy or aneuploidy is shown. Since the chromosome fragments contain essential genes, segmental aneuploidy results in inviable progeny

Using the chromosome maps described in Figs. 2 and 3, we typically enumerated the possible chromosome structures in the F1 progeny for different crossing experiments (already described or not) involving as *MAT1*-*1* partner either CBS999.97 (1-1, *re*) [10] or a QM6a *MAT1*-*1* strain with restored female fertility [9] and checked for each structure whether it contains lethal segmental aneuploidy or not. An example of the enumeration is given on Fig. 4 for a *MAT1*-*1* female fertile QM6a strain crossed with Rut-C30 strain, and the results for other crossings are shown in Table 6.

**Table 6:** Analyses of possible chromosome structures for different crossing experiments

Crossing experiment	Nb ≠ chr	Total possible structures	Non-viable	Viable	Possible viable structures different from parental ones
CBS999.97 (1-1, *re*) × CBS999.97 (1-2, *wt*) or × QM6a	2	2^2^ = 4	1	3 (75%)	1 structure with chr II fragment (D-segment) diploidy
CBS999.97 (1-1, *re*) × QM9414	4	2^4^ = 16	12	4 (25%)	1 structure with chr II fragment diploidy
					1 structure with chr II fragment diploidy and chr VI fragment diploidy
CBS999.97 (1-1, *re*) × Rut-C30	5	2^5^ = 32	23	9 (28%)	1 haploid with QM6a structure,
					1 crossed-haploid,
					4 structures with 1 chr fragment diploidy,
					1 structure with 2 chr fragment diploidy
QM6a (*MAT1*-*1, ff)* × QM6a	0	1	0	1 (100%)	None
QM6a (*MAT1*-*1, ff)* × QM9414	3	2^3^ = 8	6	2 (25%)	None
QM6a (*MAT1*-*1, ff)* × Rut-C30	3	2^3^ = 8	5	3 (38%)	1 structure with chr I fragment diploidy

The first three cases have already been experimentally described [10]. The next 3, involving a *MAT1*-*1* female fertile (ff) QM6a strain, have not yet been described. We assumed that crossing-over were possible but not in translocated parts

When crossing CBS999.97 (1-1, *re*) with industrial strains QM9414 and Rut-C30, Chuang et al. observed much more meiotic lethality (asci with no or only four viable ascopores) than when crossing with QM6a. Our theoretical results are consistent with their experimental results: while enumerating the viable chromosome structures, we observed that whereas 75% of the possible chromosome structures are viable when crossing CBS999.97 (1-1, *re*) with QM6a, only 25–28% are viable when crossing with QM9414 or Rut-C30, respectively (Table 6). For the not yet described crossings involving a *MAT1*-*1* female fertile QM6a strain, we similarly noticed that only 25 and 38% of the possible structures are viable when crossing with QM9414 and Rut-C30, respectively (Table 6). When crossing with Rut-C30, only one non-parental chromosome structure is viable (Fig. 4). When crossing with QM9414, the only possible chromosome structures are the two parental structures (Table 6). Using CBS999.97 (1-1, *re*), Chuang et al. had suggested that crossing should be used cautiously to improve industrial strains [10]. Our analysis shows that this is not due to the specific chromosome structure of this strain: using QM6a as a *MAT1*-*1* partner for crossing with industrials strains will result in almost the same meiotic lethality.

## Discussion

### Chromosome assembly

Chromosome contact data resulting from the sequencing of 3C/Hi-C libraries represent powerful information to improve or complete genome scaffolding [23]. Genome reassembly algorithms like GRAAL are based on polymer physics principles, and as such, give trustworthy, statistically sound, information about the relative position of each pair of fragments along each chromosome sequence, even when the fragments’ regions are separated by gaps which had failed to be sequenced and assembled previously. In that regard, this pipeline based on contact data outperforms current deep sequencing when trying to prove that two sequences are neighboring. For instance, GRAAL successfully integrated 63 pairs of such scaffold fragments into the QM6A reassembly which had failed to be assembled during the initial sequencing. Moreover, it was able to identify six misassemblies in the initial genome. Here, we showed that GRAAL was able to reassemble the Rut-C30 chromosomes using the QM6a sequence as a reference, and to correctly identify the six breakpoint locations specific to Rut-C30 (in addition to the misassemblies commonly found in QM6a). The pipeline can therefore identify a chromosome structure even when its sequence is not precisely known or when numerous chromosomal rearrangements occur. It could be applied with great potential to other strains, e.g., ones resulting from sexual crossing, without the need to get a sequence of these strains beforehand. Because the Rut-C30 contact map reflects the average genome organization of this strain (independently of the QM6a chromosome structure since only the reference scaffolds were used), the data could also be used for a more in-depth investigation of variations in the chromosomal contacts/interactions pattern between the two strains. However, since GRAAL is a reassembly pipeline, it does not give new information about the sequence in itself, so additional sequencing or computational work is required to fill-in the gaps between reassembled scaffolds. Misassemblies or translocation breakpoints are here identified with a ≈10 kb precision, which is sufficient here given the precise breakpoints have already been sequenced. In the case of a new strain, a chromosome walking iterative alignment of 3C-seq reads on the sequence should probably allow the identification of translocation breakpoints with the same base-pair precision.

### Centromere location and composition

Centromeres are defined as “chromosomal elements that are both necessary and sufficient for chromosome segregation” [32]. These regions display a remarkable diversity in size and structure, ranging from the so-called point 125-bp centromeres in *S. cerevisiae* to several megabases sequence of satellite DNA in plants and animals. Fungal centromeres typically range from 30 to 450 kb in size. While the point centromeres sequences seem sufficient to provide centromeric function, bigger centromeres seem to be defined epigenetically. The lack of sequence consensus even between centromeres of the same organism, and the low complexity of these AT-rich sequences have made identification and sequencing of centromeres challenging. The discovery of the centromeric histone CenH3 as the landmark of centromere regions has made chromatin immunoprecipitation the method of choice to functionally distinguish centromeric regions from other low complexity repeated regions. Here, the “Rabl” pattern of chromosomal structure in *T. reesei* observed in our previous work [23] prompted us to take advantage of the physical proximity between centromeres in this specific spatial chromosome organization for the identification of their location along the sequence [25]. The chromosomal contact data are therefore a functional proof of the centromeric nature of these sequences. As expected, the centromeric regions we determined were nearly devoid of coding sequences [21].

Interestingly, we observed in four centromeric regions (scaffolds 51, 56, 57, 58) a 7- to 10-kb long inverted repeat regions, reminiscent of inverted repeats organization found in yeasts *C. albicans, C. tropicalis*, *K. phaffii*, or *S. pombe* [31, 33–37]. To our knowledge, such an organization has not been described in filamentous fungi, as most data come from the study of *N. crassa*, whose centromeric region are 150–300 kb long and consist in degenerate transposon sequences. This raises the question of whether at least some centromeres in *Trichoderma* are sequence- or at least inverted repeat-defined, as recently hypothesized for *C. tropicalis* [37] and not only epigenetically defined. Such observation could have an influence on efforts to develop a plasmid transformation system in this fungus. Apparently, these large inverted repeat features are not unique to *Trichoderma*, as we were able to make similar observations in *Fusarium graminearum* by analyzing the latest genome sequence [41] (see Additional file 4).

### Importance of chromosome structure for analyses of crossing experiment

Knowing QM6a karyotype and chromosome translocations in some of its derivatives, we were able to predict the karyotypes of other *T. reesei* strains, from three lineages different from the NG14/Rut-C30 lineage, and to infer the possible chromosome structure in the F1 progeny for different crossing experiments involving these strains. Doing so, we managed to explain the higher meiotic lethality observed by Chuang et al. when crossing CBS999.97 (1-1,*re*) with industrial strains QM9414 and Rut-C30 compared to crossing with the natural isolate QM6a [10]. Chromosomal rearrangements resulted in chromosome structures which are not completely compatible any more in the two parents, producing lethal segmental aneuploidy in F1 progeny and conversely producing viable F1 progeny with a limited diversity in chromosome structure. This will obviously result in a limited diversity of sequence in the viable F1 progeny, since translocated fragments will undergo much less crossing-over, if any, than other parts of the genome. This imbalance may be an issue for genetics analysis-based experiments like bulk segregant analysis and for industrial strains improvement.

## Conclusions

In this work, we exploited chromosome contact data and the program GRAAL to both complete the assembly/scaffolding of the *T. reesei* reference genome, and identify its centromeres positions. That the method is robust was supported by performing the same analysis on the Rut-C30 strain, a derivative of the reference strain, which confirmed both centromeres identification and previously identified chromosome translocations in this strain. Finally, given chromosomal translocations occurred in different strain lineages of this fungus, we illustrated the importance of our data by showing predicted karyotypes of several strains and predicted consequences on crossing experiments between strains. The recent possibilities offered by strain crossings in *T. reesei* will possibly make such data and similar analyses essential in future industrial fungal research.

## Methods

### Strain and cultures

*Trichoderma reesei* Rut-C30 (strain ATCC 56765) strain was cultured in bioreactor as described previously [29].

### Construction of 3C libraries

For *T. reesei* QM6a, the construction of 3C library has already been described previously [23]. For Rut-C30 strain, the 3C library was constructed following exactly the same protocol and restriction enzyme (DpnII).

### GRAAL assembly

Genome (Re)Assembly Assessing Likelihood from 3D (GRAAL) is an algorithm which uses chromosome conformation capture (3C) data to rescaffold contigs and improve genome assembly [23]. Briefly, the original genome is first split into bins containing the same number of restriction fragments (a restriction fragment is a genome region between two restriction sites of the enzyme used for the 3C library construction), then the reads from the 3C library are mapped onto these bins so as to compute an initial contact matrix, each entry therein representing the contact frequency between each bin pair and bins being ordered along the initial genome assembly. This matrix shows contact discrepancies since the original genome is not fully assembled. Finally, GRAAL reorders the bins so as to get the most likely matrix based on what contact frequency distribution is expected from chromosomes according to a standard polymer physics model [42]. The *T. reesei* QM6a chromosome sequence we previously published is an example of the raw output from the algorithm.

### Manual corrections

Several GRAAL computations were performed with different bin sizes to assess the assembly’s robustness. Then manual corrections were performed to go beyond the limitations of GRAAL and other reassembly programs. Since scaffolds were split into bins with the same number of restriction fragments, scaffold ends were too small (sequencing coverage too low) to be included in the computation, so were lost in the raw output sequence. We manually added them so as to get the entire scaffolds in the reassembly. When a scaffold is misassembled in the original genome, GRAAL is able to find the splitting location at an accuracy depending on the size of bins involved in the splitting (around 10–50 kb depending on the definition of the bins, and on the location of the restriction sites). We checked the sequence around the splits and most of the time we noticed nearby the presence of ≈1 kb NNN sequences, so we manually corrected the split location to be consistent with this gap location. Reassembly programs like GRAAL easily reorder bins using contact data, but they may fail in finding the correct bin orientation, so many bins were switched (by comparison with the neighboring bins from the same original scaffold) in the raw output sequence. We manually corrected them to get the scaffolds as in the original assembly without switching bins. However, some scaffolds were too small to get a reliable orientation, in this case, we arbitrarily chose the forward direction for the sequence available in Additional file 2. Seven telomere repeats were identified in the original sequence [13] and six of them were assembled in the chromosomes, as noticed previously [26] although they were not at chromosome ends in the raw output sequence. We checked their presence at chromosome ends, and used them three times to identify the correct bin directions (for scaffold 45, 46, and 64 in chromosomes III, V, and IV, respectively). As for scaffold 31 on chromosome VI, we deleted 7 kb at the 3′ end because they were duplications of the telomere sequence. Around 20–30 bins (<4% of the total number of bins) had not been reassembled because their signature in the contact matrix was not strong enough for GRAAL. We manually checked the contact matrix and reassembled these bins in the final sequence depending on their contact signature (telomere, centromere, standard). Finally, the gene annotation from the JGI (gtf file for the Filtered Models set of genes, [43]) was mapped to the reassembled sequence in order to get the coordinates of the 9129 genes on the chromosomes.

### Centromere positions

Centromere positions along the chromosomes have been manually identified using the contact data (see Additional file 7 for raw data contact frequencies over the entire genome). Because of their Rabl organization, centromeres have stronger interaction with each other than with their neighboring sequences.

### Gene enrichment analysis

To calculate the enrichment in genes close to the centromeres, we used the gene annotations (GO terms and EC numbers) from the JGI [43] and from the FungiPath database [44–46], and performed the enrichment analysis with the Pathway Tools software [47]. A 50-kb window was defined around the centromeres, which resulted in a set of 238 genes (2.6% of the genome).

## Additional files

**Additional file 1.** Details on QM6a reassembly.

**Additional file 2.** QM6a reassembly sequence in fasta format (7 chromosomes + unassembled scaffolds).

**Additional file 3.** Annotation file describing the location on the chromosomes of i) the original scaffolds, ii) the centromeres and iii) the 9129 genes from the JGI Filtered Models set of genes.

**Additional file 4.** Identification of inverted repeats in *T. reesei* and *F. graminearum* centromeres.

**Additional file 5.** Details on Rut-C30 reassembly.

**Additional file 6.** List of gene markers used on Figs. 2 and 3, with their names, IDs, locations on scaffolds and chromosomes, and functional annotations.

**Additional file 7.** Raw data contact frequencies over the entire genome.

## Abbreviations

PFGE: pulse-field gel electrophoresis
3C: chromosome conformation capture
CDS: coding sequence
