# Correction to: Proximity ligation scaffolding and comparison of two *Trichoderma reesei* strains genomes

**DOI:** 10.1186/s13068-018-1161-5

**Published:** 2018-06-13

**Authors:** Etienne Jourdier, Lyam Baudry, Dante Poggi-Parodi, Yoan Vicq, Romain Koszul, Antoine Margeot, Martial Marbouty, Frédérique Bidard

**Affiliations:** 1 IFP Energies nouvelles, 1 et 4 Avenue de Bois-Preau, 92852 Rueil-Malmaison, France; 2 Groupe Régulation Spatiale des Génomes, Department Genomes and Genetics, Institut Pasteur, 75015 Paris, France; 3 UMR 3525, CNRS, 75015 Paris, France

## Correction to: Biotechnol Biofuels (2017) 10:151 10.1186/s13068-017-0837-6

Following publication of the original article [1], the authors reported a problem in the drawing of Rut-C30 chromosome III in Fig. 2b of the original article [1]. The two fragments of chromosome I inserted inside chromosome III should be swapped, and the direction of the fragment containing rim101 and cel1a genes should be inverted. This reversed insertion indicates that at least 2 rearrangements occurred simultaneously, so the possible scenario proposed in Fig. 2c of the original article was inaccurate. The corrected Fig. 2 with modified panels b and c is available in this erratum. The detailed description of Rut-C30 assembly in the Additional file 5 of the original article is correct.

**Fig. 2:**
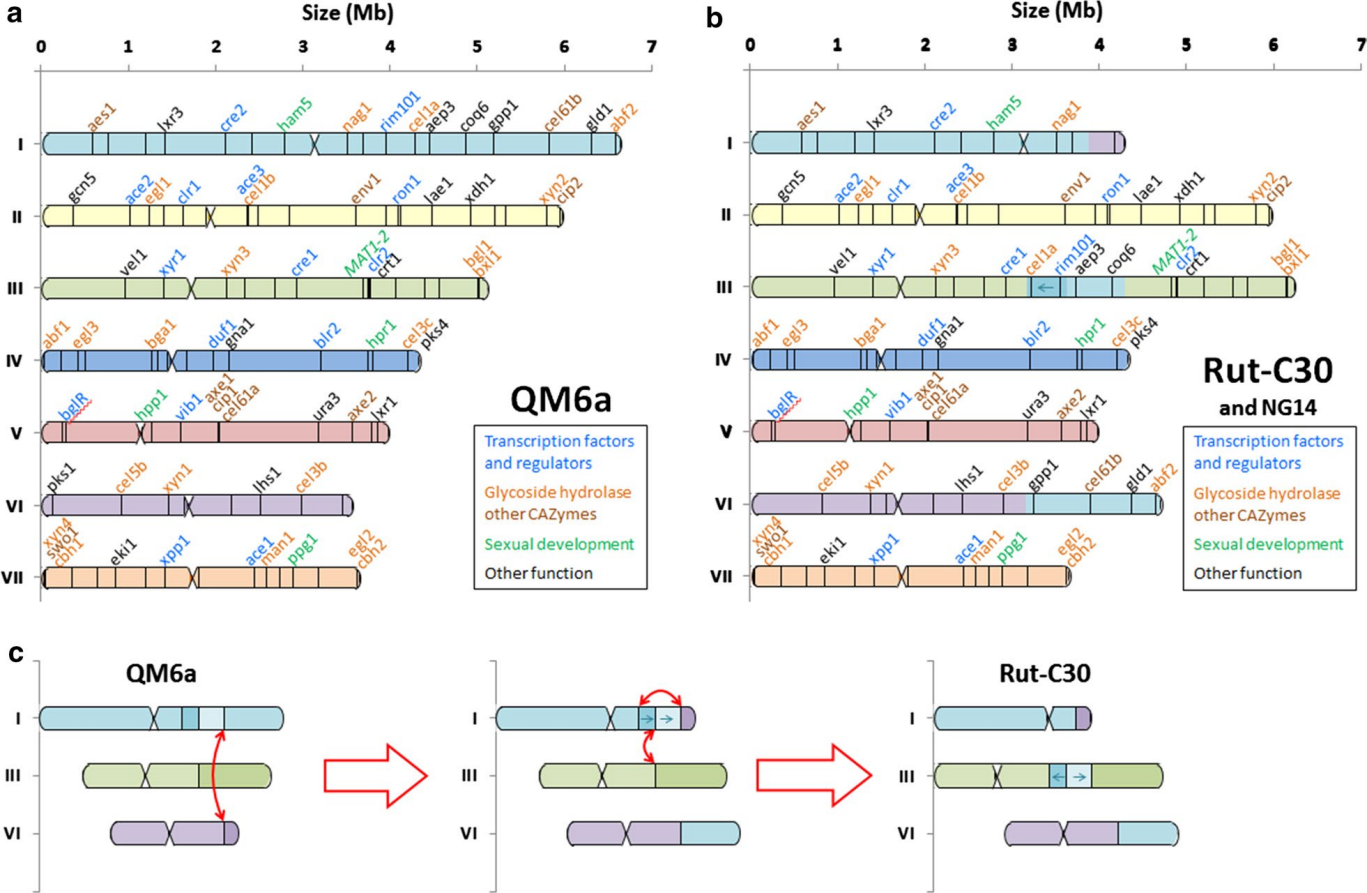
Chromosome maps of *T. reesei* QM6a and Rut-C30 strains. Chromosome maps of *T. reesei* QM6a (**a**) and Rut-C30 and NG14 (**b**) strains were identified by reassembly of the JGI reference genome using 3C sequencing data for each strain. For Rut-C30 map, the *colors* of chromosome fragments are consistent with their *colors* in QM6a map to clearly show chromosomal rearrangements. Some emblematic genes were chosen along the sequence to be used as location markers (list available in Additional file 6). The Rut-C30 85 kb deletion event on chr. VI is shown by the lack of pks1 gene. Centromere locations are shown by restricted width. **c** Possible scenario (among others) from QM6a to Rut-C30

The authors also noticed two mistakes in chromosome numbering in the description of these translocations. The correct description is

- “The three translocations resulted finally in the right arm of chromosome I (3′ end of scaffold 48 and main fragment of scaffold 5: 1.63 Mb and 442 genes in total), to be swapped with the right arm of chromosome **VI** (3′ end of scaffold 22: 402 kb and 114 genes). But also in two fragments of chromosome I (one with a fragment of scaffold 4, and the other one with another fragment of scaffold 4, scaffold 49 and a small fragment of scaffold 48) to be inserted head to foot in the middle of the chromosome **III** (1.13 Mb and 310 genes in total for both fragments).”
