# Supplementary material for: Proximity ligation scaffolding and comparison of two Trichoderma reesei strains genomes
Source: Biotechnol Biofuels. 2017 Jun 12;10:151. doi: 10.1186/s13068-017-0837-6 (PMC5469131; doi:10.1186/s13068-017-0837-6)
Supplement: Supplementary file 1 — Additional file 1. Details on QM6a reassembly. [file 13068_2017_837_MOESM1_ESM.pdf]

T. reesei QM6a reassembly

65 scaffolds from the JGI reference genome (33.3Mb - 99.5% of the genome) have been reassembled in 7 chromosomes, as follows :

CHR I

scaffold		start	end	direction	
7	full	1	1 429 972	-1	
12	full	1	1 022 062	-1	
43	full	1	74 996	-1	
21	full	1	576 034	-1	
55	full	1	33 670	?	centromere - direction uncertainty
4	full	1	1 832 615	1	
49	full	1	46 304	-1?	direction uncertainty
48	full	1	48 367	1	
5	fragment	1	1 583 115	-1	split location identified by Ns gap
		size :	**6 647 935**	(with 100bp Ns spacers between each scaffold)	

CHR II

scaffold		start	end	direction	
31	fragment	1	224 034	-1	telomere repeats (4 duplications of the telomere sequence have been deleted, 7kb)
41	full	1	80 626	-1	
25	full	1	439 677	1	
68	full	1	10 734	1	
10	full	1	1 156 739	-1	
66	full	1	11 200	?	centromere - direction and order uncertainty
59	full	1	18 517	?	centromere - direction and order uncertainty
8	full	1	1 408 331	1	
34	full	1	166 473	-1	
26	full	1	433 400	1	
14	full	1	861 070	-1	
23	full	1	512 080	-1	
54	full	1	34 758	1?	direction uncertainty
36	full	1	136 855	-1	
27	full	1	433 262	-1	
28	fragment	367 024	407 093	?	split location identified by Ns gap - direction uncertainty
67	full	1	11 021	1?	direction uncertainty
		size :	**5 980 447**	(with 100bp Ns spacers between each scaffold)	

CHR III

scaffold		start	end	direction	
45	full	1	65 952	-1	telomere repeats OK
69	full	1	10 696	?	direction uncertainty
35	full	1	152 537	1	
32	full	1	230 370	-1	
11	full	1	1 155 933	-1	
40	full	1	89 857	1	
					centromere position (no centromere scaffold reliable assembled)
2	fragment	154 748	2 007 204	-1	split location uncertainty (154 748 was chosen here after alignment with fragment 1-98434)
2	fragment	1	98 434	-1	split location identified by Ns gap
6	full	1	1 455 714	-1	telomere repeats OK
		size :	**5 112 650**	(with 100bp Ns spacers between each scaffold)	

CHR IV

scaffold		start	end	direction	
64	full	1	14 482	1	telomere repeats
19	full	1	663 018	-1	
17	full	1	797 352	1	
56	full	1	32 194	?	centromere - direction uncertainty
20	full	1	629 213	-1	
1	fragment	2 981 735	3 756 989	1	split location identified by Ns gap
5	fragment	1 584 116	1 729 360	1	split location identified by Ns gap
13	full	1	891 309	-1	
2	fragment	99 435	154 747	-1	split location uncertainty (around 152 to 158 kb, 154747 chosen after alignment of the 2 other fragments)
38	full	1	125 035	-1	
33	full	1	207 997	-1	
		size :	**4 337 413**	(with 100bp Ns spacers between each scaffold)	

CHR V

scaffold		start	end	direction	
46	full	1	62 252	1	telomere repeats
30	full	1	247 268	1	
42	full	1	78 584	1	
53	full	1	36 593	1	
18	full	1	685 578	1	
61	full	1	15 406	-1?	centromere - direction and order uncertainty
60	full	1	15 714	1?	centromere - direction and order uncertainty
28	fragment	1	366 023	-1	split location identified by Ns gap
1	fragment	1	2 471 118	1	telomere repeats - split location identified by Ns gap and presence of telomere repeats
		size :	**3 979 336**	(with 100bp Ns spacers between each scaffold)	

CHR VI

scaffold		start	end	direction	remarks
47	full	1	50 543	-1?	direction uncertainty
15	full	1	837 556	1	
44	full	1	66 247	-1	
62	full	1	15 337	-1?	direction uncertainty
1	fragment	2 471 169	2 980 271	-1	split location identified by Ns gap and presence of telomere repeats on the other side
50	full	1	45 663	-1	
37	full	1	132 540	1	
51	full	1	43 169	?	centromere - direction uncertainty
39	full	1	105 148	1	
9	full	1	1 219 543	-1	
22	full	1	541 456	1	

size : 3 567 305 (with 100bp Ns spacers between each scaffold)

CHR VII

scaffold		start	end	direction	
29	full	1	382 182	-1	
24	full	1	501 049	-1	
16	full	1	824 923	-1	
52	full	1	41 083	?	centromere - direction uncertainty
3	full	1	1 910 749	-1	

size : 3 660 386 (with 100bp Ns spacers between each scaffold)

>scaffold_57

scaffold		start	end	direction	
57	full	1	25 756	1	centromere signature but not reliably assembled

>scaffold_58

scaffold		start	end	direction	
58	full	1	21 040	1	centromere signature but not reliably assembled

>scaffold_63

scaffold		start	end	direction
63	full	1	14 539	1

>scaffold_65

scaffold		start	end	direction	
65	full	1	12 580	1	centromere signature but not reliably assembled

>scaffold_70

scaffold		start	end	direction
70	full	1	8 513	1

>scaffold_71

scaffold		start	end	direction
71	full	1	6 846	1

>scaffold_72

scaffold		start	end	direction
72	full	1	6 811	1

>scaffold_73

scaffold		start	end	direction
73	full	1	6 421	1

>scaffold_74

scaffold		start	end	direction
74	full	1	5 890	1

>scaffold_75

scaffold		start	end	direction
75	full	1	5 683	1

>scaffold_76

scaffold		start	end	direction
76	full	1	5 459	1

>scaffold_77

scaffold		start	end	direction
77	full	1	5 371	1

>scaffold_78

scaffold		start	end	direction
78	full	1	5 154	1

>scaffold_79

scaffold		start	end	direction
79	full	1	4 691	1

>scaffold_80

scaffold		start	end	direction
80	full	1	4 619	1

>scaffold_81

scaffold		start	end	direction
81	full	1	4 614	1

>scaffold_82

scaffold		start	end	direction
82	full	1	4 370	1

>scaffold_83

scaffold		start	end	direction
83	full	1	3 796	1

>scaffold_84

scaffold		start	end	direction
84	full	1	3 468	1

>scaffold_85

scaffold		start	end	direction
85	full	1	3 089	1

>scaffold_86

scaffold		start	end	direction
86	full	1	3 000	1

>scaffold_87

scaffold		start	end	direction
87	full	1	2 158	1
