# Supplementary material for: Proximity ligation scaffolding and comparison of two Trichoderma reesei strains genomes
Source: Biotechnol Biofuels. 2017 Jun 12;10:151. doi: 10.1186/s13068-017-0837-6 (PMC5469131; doi:10.1186/s13068-017-0837-6)
Supplement: Supplementary file 4 — Additional file 4. Identification of inverted repeats in T. reesei and F. graminearum centromeres. [file 13068_2017_837_MOESM4_ESM.pdf]

Additional file 4 : identification of inverted repeats in *T. reesei* and *F. graminearum* centromeres

In 4 cases (scaffolds 51 (chr I), 56 (chr IV), 57 and 58), we observed in centromere scaffolds an inverted repeat structure with a central core region of 1 to 2 kb surrounded by an inverted repeat of 2.5 to 5 kb (Figure S2A). This structure seems quite similar to the centromere structure of *S. pombe* [1–3], *Candida tropicalis* [4] and *Komagataella phaffii* (formerly *Pichia partoris*) [5] (Figure S2B).

We annotated these sequences "mid" for the central cores and , "LR" for the left repeat, and "RR" for the right repeat, consistently with *C. tropicalis* and *K. phaffii* [4, 5], followed by the chromosome or scaffold number (Figure S2A below). The LR4 and RR4 sequences of the inverted repeat of chr IV centromere (scaffold 56) share 92% identity on 4kb without any gaps. In the 3 other cases, the LR and RR sequences share ≈58% identity but with large gaps (identity reaches 84 to 92% while excluding gaps).

While these observations could result from a misassembly of these AT-rich regions, they suggest that centromere structure in *Trichoderma* is significantly different from what is described in *Neurospora* and other filamentous fungi [6], and share some similarities with structures observed in *Taphrinomycotina* and *Saccharomycetales*.

Moreover, using the latest *Fusarium graminearum* genome release [7], we observed undescribed similar inverted repeats in the centromeres of *F. graminearum* chromosomes 1 and 2 (Figure S2B).

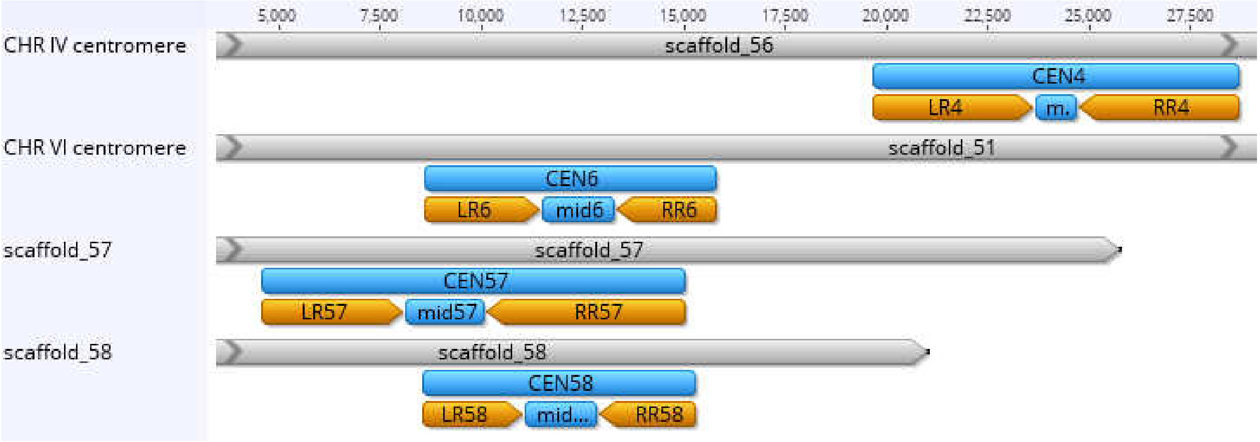

Figure S2A: Inverted repeats found on centromere-involved scaffolds
Four similar structures with a central core (mid) region surrounded by an inverted repat (LR and RR) sequences were identified on 4 scaffolds involved in *T. reesei* centromeres (scaffold 56 in chr IV centromere, scaffold 51 in chr VI centromere, and scaffolds 57 and 58 with centromere signature but not assembled).

Figure S2B: Sequence alignment of centromeres on themselves

Core centromere sequences (containing LR, mid and RR sequences) have been aligned against themselves using the LASTZ software [8, 9] with default parameters, in order to show the inverted repeats. This figure includes the 4 sequences from *T. reesei*, and arbitrary chosen sequences from *S. pombe, K. phafii, C. tropicalis* and *F. graminearum*.

Strain and location	Sequence alignment on itself using LASTZ
T. reesei CEN4<br><br>9,130 bp<br><br>Scaffold_56:19,652-28,781	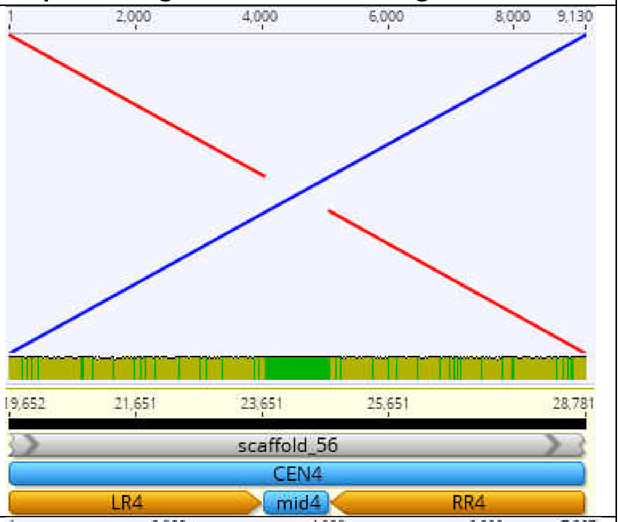
T. reesei CEN6<br><br>7,287 bp<br><br>Scaffold_51:8,571-15,857	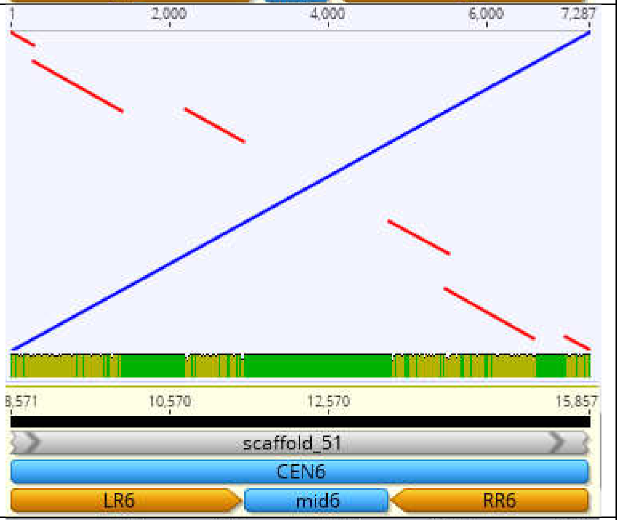
T. reesei CEN57<br><br>10,548 bp<br><br>Scaffold_57:4,556-15,103	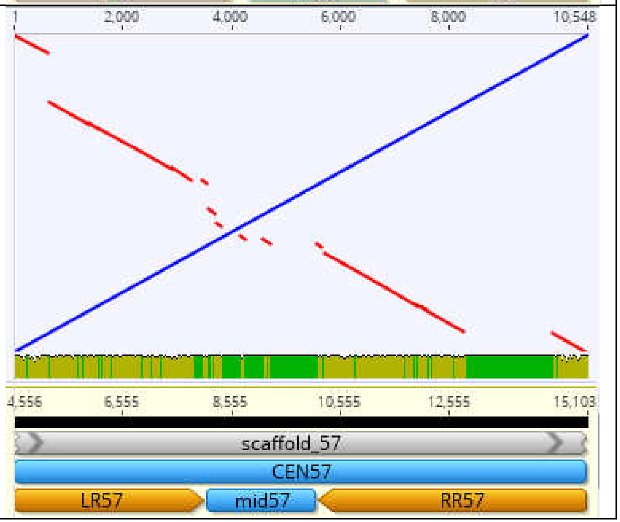

T. reesei CEN58<br><br>6,812 bp<br><br>Scaffold_58:8,532-15,343	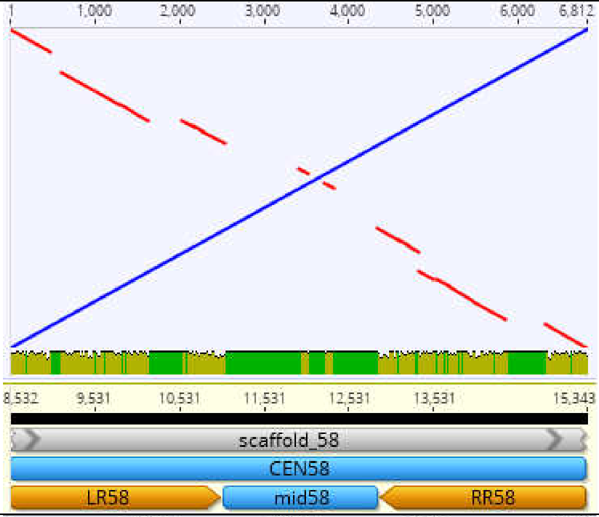
Schizosaccharomyces pombe CEN2<br><br>41,139 bp<br><br>Chromosome II: 1,602,264-1,6447,747	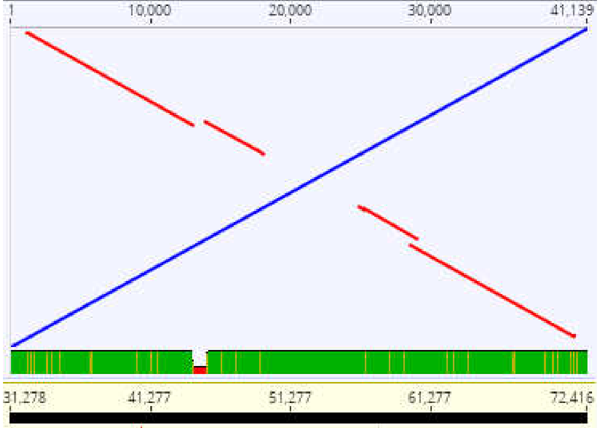
Komagataella phaffii (Pichia pastoris) CEN2<br><br>6,655 bp<br><br>Chromosome 2 (FR839629.1): 843,845-850,499	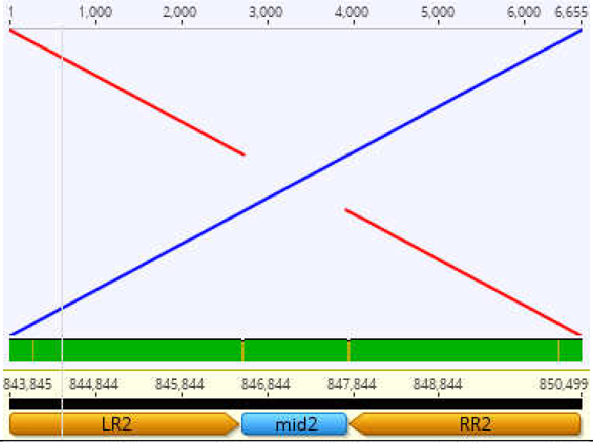
Candida tropicalis CEN5<br><br>10b113 bp<br><br>Supercontig3.5 (GG692399.1): 718,785-728,897	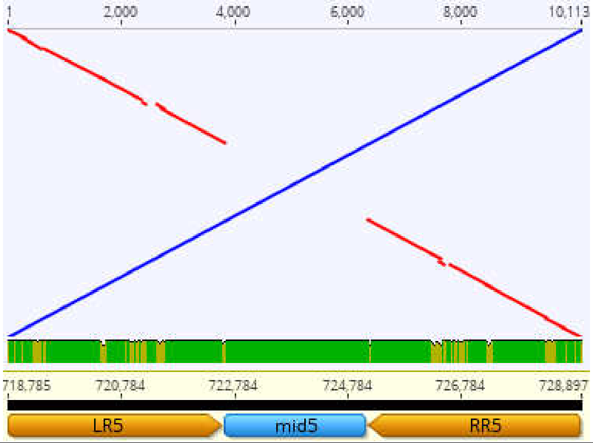

<table>
<tr>
<td>Fusarium graminearum CEN1

9,818 bp

Chromosome 1:
8,976,756-8,986,573</td>
<td>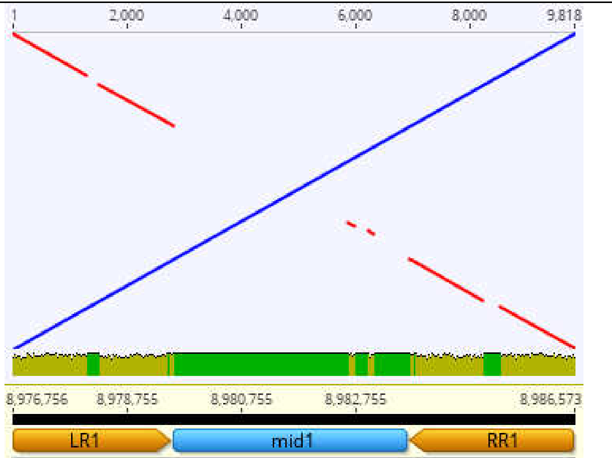
</td>
</tr>
<tr>
<td>Fusarium graminearum CEN2

11,796 bp

Chr2:
3,288,357-3,330,152</td>
<td>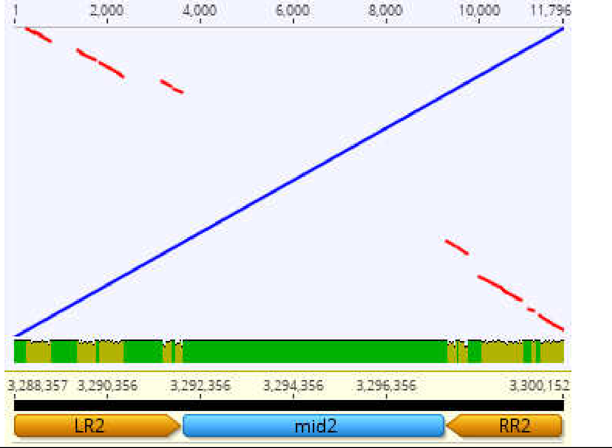
</td>
</tr>
</table>

References

1. Wood V, Gwilliam R, Rajandream M-A, Lyne M, Lyne R, Stewart A, et al. The genome sequence of *Schizosaccharomyces pombe*. Nature 2002;415:871–80. doi:10.1038/nature724.
2. Nakaseko Y, Adachi Y, Funahashi S-i, Niwa O, Yanagida M. Chromosome walking shows a highly homologous repetitive sequence present in all the centromere regions of fission yeast. The EMBO Journal 1986;5:1011–21.
3. Fishel B, Amstutz H, Baum M, Carbon J, Clarke L. Structural organization and functional analysis of centromeric DNA in the fission yeast *Schizosaccharomyces pombe*. Molecular and Cellular Biology 1988;8:754–63.
4. Chatterjee G, Sankaranarayanan SR, Guin K, Thattikota Y, Padmanabhan S, Siddharthan R, Sanyal K. Repeat-Associated Fission Yeast-Like Regional Centromeres in the Ascomycetous Budding Yeast *Candida tropicalis*. PLoS Genet 2016;12:e1005839. doi:10.1371/journal.pgen.1005839.
5. Coughlan AY, Hanson SJ, Byrne KP, Wolfe KH. Centromeres of the Yeast *Komagataella phaffii* (*Pichia pastoris*) Have a Simple Inverted-Repeat Structure. Genome Biology and Evolution 2016;8:2482–92. doi:10.1093/gbe/evw178.
6. Smith KM, Galazka JM, Phatale PA, Connolly LR, Freitag M. Centromeres of filamentous fungi. Chromosome Research 2012;20:635–56. doi:10.1007/s10577-012-9290-3.

7. King R, Urban M, Hammond-Kosack MCU, Hassani-Pak K, Hammond-Kosack KE. The completed genome sequence of the pathogenic ascomycete fungus *Fusarium graminearum*. BMC Genomics 2015;16:1–21. doi:10.1186/s12864-015-1756-1.
8. Schwartz S, Kent WJ, Smit A, Zhang Z, Baertsch R, Hardison RC, et al. Human–Mouse Alignments with BLASTZ. Genome Research 2003;13:103–7. doi:10.1101/gr.809403.
9. Harris RS. Improved pairwise alignment of genomic DNA [PhD thesis]: Pennsylvania State University; 2007.
